# Clinical characteristics and risk factors of ovarian reserve decreases in women with Crohn’s disease: a case-control study

**DOI:** 10.1186/s13048-023-01112-6

**Published:** 2023-02-07

**Authors:** Peng Xiang, Jia-yin Yao, Xiao-lan Li, Min Zhang, Pin-jin Hu, Min Zhi

**Affiliations:** 1 Department of Gastroenterology, the Sixth Affiliated Hospital, Sun Yat-sen University, No.26, Erheng Road, Yuancun, Tianhe District, Guangzhou, 510655 People’s Republic of China; 2 Guangdong Provincial Key Laboratory of Colorectal and Pelvic Floor Diseases, the Sixth Affiliated Hospital, Sun Yat-sen University, Guangzhou, 510655 People’s Republic of China; 3 Department of Reproductive Medicine Center, the Sixth Affiliated Hospital, Sun Yat-sen University, Guangzhou, 510655 People’s Republic of China

**Keywords:** Crohn’s disease, Anti-Müllerian hormone, Ovarian reserve

## Abstract

**Background:**

Crohn’s disease (CD), often occurring in women of child-bearing age, can decline the fertility rate. However, whether it reduces ovarian reserve has been rarely reported. This study aimed to evaluate the ovarian reserve in women with CD from the perspective of anti-Müllerian hormone (AMH), and explore the factors that can decrease ovarian reserve.

**Methods:**

A case-control retrospective study was designed. We analyzed the AMH levels in a total of 135 CD women and 878 healthy controls. Through propensity score matching, the subjects were assigned in a ratio of 1:3 to CD group (*n* = 121) and control group (*n* = 324). Both groups shared similar basic characteristics, like age, body mass index and smoking status. Serum AMH levels were measured by chemiluminescence.

**Results:**

The AMH level in the CD group was significantly lower than that in the control group (2.17 ± 2.23 μg/L vs 3.95 ± 2.01 μg/L, 95%CI [1.34-2.21], *P* < 0.001). In both groups, the AMH levels decreased as age increased, but without between-group difference in the decreasing rate (*P* = 0.639). Multivariate analysis showed that age > 30 years (OR, 2.905; 95%CI [1.053-8.531], *P* = 0.017), disease activity (OR,4.314; 95%CI [1.561-12.910], *P* = 0.002) and thalidomide use (OR,12.628; 95%CI [4.351 -42.820], *P* < 0.001) were independent risk factors associated with decreased ovarian reserve (AMH<1.1μg/L).

**Conclusion:**

Ovarian reserve is lower in CD women than in healthy women. Age, CD activity and medication of thalidomide are risk factors that can aggravate the decline of ovarian reserve.

## Introduction

Crohn’s disease (CD), a chronic non-specific inflammatory bowel disease, often sets on in women aged 20-35 years, a high time of child-bearing [1]. CD may exert negative effects on women’s fertility and pregnancy [2]. It has reported that the fertility of CD women declines, meanwhile the risk of infertility rises [3]. Multiple factors contribute to fertility decline in CD women. (1) Inflammatory response poses deleterious effects on the function of organs in the pelvic cavity. For example, salpingitis, ovaritis and pelvic surgery may cause infertility, and medication in treating these diseases may also damage ovarian function [4, 5]. (2) CD activity, surgery, pain and sexual dysfunction can lead to sexual inactivity and subsequent infertility [6]. (3) CD women may refuse fertility for fear of the heritability of CD, or the negative effects of CD on pregnancy and fetus [7]. About 14-18% of CD women initiatively give up pregnancy, with a proportion significantly higher than the 6% in healthy women [8, 9]. (4) CD women may experience psychological disorders, like anxiety and depression, with a high incidence doubling that in the healthy population. These disorders further impair sexual function and consequent fertility. These four aspects can explain the high infertility rate in CD women [10].

The pregnancy and fertility of CD women can be indirectly evaluated through pregnancy outcomes, such as the number of infants or the delay of fertilization in the married women. In addition, some research findings are contradictory. In fact, infertility is sometimes a result of initiative selection or national policy. Pregnancy outcomes cannot offer an objective reflection on the fertility and ovarian reserve in CD women.

Ovarian reserve, judged by the number and quality of follicles remaining in the ovary, can be used to provide reliable information about female fertility. As the ovarian reserve decreases, the ability of the ovary to produce oocytes and the quality of follicles fall, probably followed by infertility. Ovarian reserve can be estimated through various hormones, such as follicle stimulating hormone (FSH), estradiol (E2), luteinizing hormone (LH) and inhibin B [11]. However, the efficiency of these indexes is always restricted. Anti-Müllerian hormone (AMH) is released by preantral or antral follicles. In the peripheral blood, AMH is the follicle-released substance that can be first detected. The level of AMH fluctuates slightly during the menstrual cycle, making it successfully detected at any time point. Compared to other hormones, AMH is more convenient and accurate in evaluating ovarian reserve [12]. Low AMH can predict decreased ovarian reserve. As usual, an AMH level < 1.1μg/L indicates a low ovarian reserve [13, 14].

In this study, we retrospectively analyzed the AMH levels in CD women and healthy control, aiming to find out CD-related factors affecting ovarian reserve.

## Materials and methods

### Design and subjects

A retrospective analysis was designed. Recruited were patients treated for CD at the Inflammatory Bowel Disease Center of the Sixth Affiliated Hospital, Sun Yat-sen University from July 2017 to December 2021. This case-control study was approved by the Ethics Committee of the Sixth Affiliated Hospital, Sun Yat-sen University (2017ZSLYEC-077). Inclusion criteria: (1) women aged 20-40 years. (2) CD diagnosed within previous 6 months according to medical history, endoscopic, imaging and pathological features, and European Crohn’s and Colitis Organization (ECCO) consensus guidelines.

Data of healthy and aged-matched controls were collected from demographic bank of the Reproductive Medicine Center at our hospital. The database was established by the hospital for the epidemiological survey of ovarian function in women of reproductive age. The control were selected from women without known diseases and who had never received any drug. In addition, women must have no Crohn’s disease.

Exclusion criteria for two groups included: (1) previous surgery of ovaries; (2) polycystic ovarian syndrome; (3) history of ovarian cancer; (4) endocrine and metabolic diseases; (5) hepatic, renal and cardiac dysfunction; (6) previous use of cytotoxic drugs; (7) history of pelvic radiotherapy; (8) oral contraceptives; (9) history of malignant tumors. This study was approved by the Ethics Committee of the Sixth Affiliated Hospital, Sun Yat-sen University. All subjects provided written informed consent.

## Methods

Data about age, height, weight, body mass index (BMI), smoking, disease duration, location, disease behavior, previous medical therapies, C-reactive protein (CRP), hemoglobin, albumin and AMH were collected from medical records or demographic bank of the Center. The serum AMH level detection methods for the both groups were consistent. The samples were detected with automatic chemiluminescence method in the same center using the kit from the same company (AMH Immunotech S.A.S a Beckman Coulter Company, France).

### Statistical analysis

All analyses were performed on SPSS 22.0. According to the technique of propensity score matching, the basic data were matched in a ratio of 1:3. Common statistical tests included Kolmogorov-Smirnov test (KS test) and Shapiro-Wilk test (SW test). When the *p* value of test results was less than 0.05, we considered that the data were not in the normal distribution. Continuous variables were expressed as mean ± standard deviation. The nonpaired data in normal distribution were analyzed by *t* test, otherwise by Wilcoxon2-sample test. Categorical variables were expressed as absolute value and percentage, and subjected to Fisher’s exact test or Yates’ chi-square test. One-way analysis of variance was performed for screening risk factors. Those with *p* < 0.2 were submitted to binary logistic regression analysis. All analyses were two-tailed and *P* < 0.05 was considered as statistically significant.

## Results

### Basic data

A total of 135 CD women and 878 healthy women were enrolled. After propensity score matching, 121 CD women were assigned to the Crohn’s group and 324 healthy women to the control group, with similar age, BMI and smoking status (Table 1).

**Table 1:** Age, BMI Score, Smoker Status in Crohn’s and Control Group

	Before PSM		*P*	After PSM		*P*
	CD (*n* = 135)	Control (*n* = 878)		CD (*n* = 121)	Control (*n* = 324)	
Age, mean (SD), Y	28.31(5.49)	30.54(4.19)	< 0.001	28.75(15.49)	29.34(3.97)	0.212
BMI, score, mean (SD)	18.91(2.61)	21.76(3.04)	< 0.001	19.33(2.37)	19.68(2.05)	0.119
Smoke, n (%)	5 (3.70)	25 (2.85)	0.583	4(3.31)	9(2.78)	0.756
Patient with children, n (%)	–	–	–	73(60.33)	226(69.75)	0.069
Patient with miscarriage, n (%)	–	–	–	16(13.22)	31(9.57)	0.298

### AMH levels in two groups

**Fig. 1:**
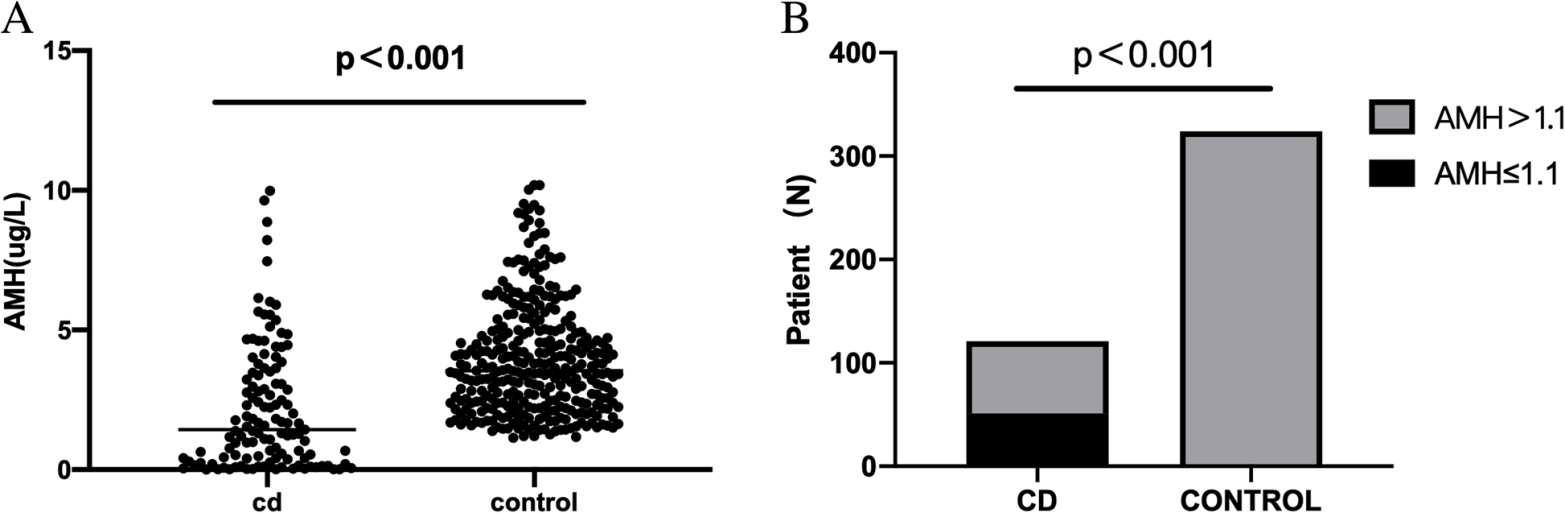
Comparison of AMH between CD patients and control

The AMH level in the CD group was significantly lower than that in the control group (2.17 ± 2.23 μg/L vs 3.95 ± 2.01 μg/L, 95%CI [1.34 to 2.21], *P* < 0.001) (Fig. 1A). The proportion of women with decreased ovarian reserve (AMH ≤ 1.1μg/L) was higher than that in the control group (42.15% vs 0%, *p* < 0.001) (Fig. 1B).

### Association between AMH and age

**Fig. 2:**
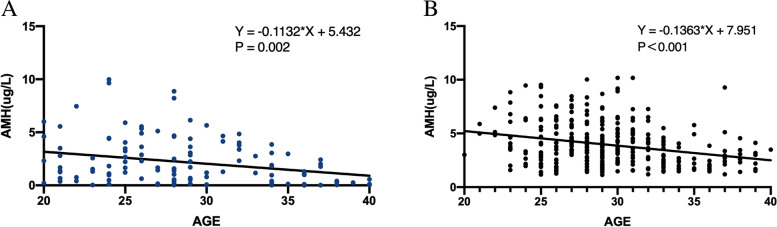
Relationship between AMH and age in CD and control group

In both groups, the AMH level decreased as age increased. The slope of the regression line was − 0.11 in the CD group and − 0.13 in the control group, without statistical difference (*P* = 0.639) (Fig. 2).

### Factors affecting ovarian reserve

**Fig. 3:**
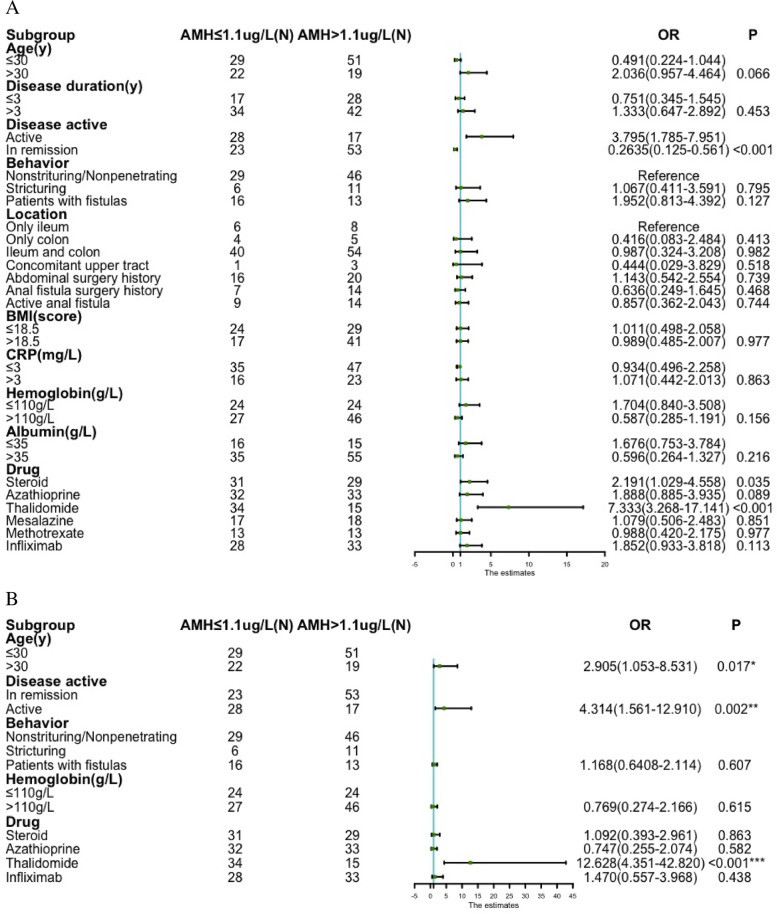
Risk factors associated with decreased ovarian reserve

In the CD group, 51 (42.15%) of patients presented decreased ovarian reserve. As found in analyses of age, disease activity, behavior and medication history, the decreased ovarian reserve was associated with age > 30 years (OR, 2.036; 95%CI[0.957-4.464], *P* = 0.066), disease activity (OR, 3.795; 95%CI[1.785-7.951], *P* < 0.001), penetrating lesion (OR, 1.952; 95%CI[0.813-4.392], *P* = 0.127), anemia (Hg ≤ 110 g/L) (OR, 1.704; 95%CI[0.840-3.508], *P* = 0.156), steroids (OR, 2.191; 95%CI[1.029-4.558], *P* = 0.035), azathioprine (OR, 1.888; 5%CI[0.885-3.935;], *P* = 0.089), thalidomide (OR, 7.333; 95%CI[3.268-17.141],*P* < 0.001), infliximab (OR, 1.852; 95%CI[0.933-3.818], *P* = 0.113) (Fig. 3A). Variables with *P* < 0.2 were included in the binary logistic regression analysis. We found that age > 30 years (OR, 2.905; 95%CI[1.053-8.531], *P* = 0.017), disease activity (OR, 4.314; 95%CI[1.561-12.910], *P* = 0.002), and thalidomide use (OR, 12.628; 95%CI[4.351-42.380], *P* < 0.001) were independent risk factors for decreased ovarian reserve (Fig. 3B).

## Discussion

CD often sets on in a child-bearing age [1]. It remains a controversy whether CD impairs fertility of women. Studies have shown a lower fertility rate in CD women than in the healthy [15]. In our study, the number of children in the CD group were lower than the control group, and the miscarriage rate was higher, but no significant difference was found. It may be related to several factors. For example, some of the women with Crohn’s disease have had children before diagnosis, some may have a delayed fertilization plan, or some may be unwilling to be fertilized. Besides, the disease activity, medication history and surgery may also influence the fertility rate. Meanwhile, as the fertilization delays, the ovarian reserve declines. In addition, CD may also involve the ovarian ducts and the ovaries, the inflammation of which also damages female fertility [16, 17]. However, changes in fertility rate of patients could not fully represent the true ovarian reserve function. Therefore, it is a clinical urgency to define the association of CD with female fertility, as well as the risk factors of fertile failure [18, 19]. This information can help CD women to design an optimal strategy for pregnancy.

A lower AMH level predicts a poorer ovarian reserve or a weaker response to ovarian stimulation. A study has verified the accuracy of AMH in predicting the ovarian reserve in females with lupus erythematosus, indicating that AMH can be used to evaluate the toxicity in sexual glands. Therefore, AMH was introduced into the present study to judge the ovarian reserve and potential fertility of females with CD.

In this study, the AMH level in CD women was significantly lower than that in the healthy (2.17 ± 2.23μg/L vs 3.95 ± 2.01μg/L; 95%CI [1.34 to 2.21], *P* < 0.001), so was the incidence of decreased ovarian reserve (AMH ≤ 1.1 μg/L) (42.15% vs 0%, *P* < 0.001). These findings verify that the ovarian reserve declines in CD women, thus explaining why CD women show a lower fertility rate than the healthy [17, 20].

Ovarian reserve falls as age increases, which is also proven in the present study. In both groups, the AMH levels decreased with age, but their decreasing rate showed no significant between-group difference. Subgroup analysis further demonstrated that age > 30 years (OR, 2.905; 95%CI [1.053-8.531], *P* = 0.017) was an independent risk factor of decreased ovarian reserve in CD women, a finding that supports the profile of AMH in these patients. Freour [21] et al. reported that AMH levels were significantly lower in CD women under 30 years old, but remained comparable in those over 30 years old. This difference may be attributed to the fact that their study only chose the patients with quiescent disease, and an IVF population with a normal ovarian reserve status as the control group, who can be considered different from the general population. For CD patients who delay their ferritization plan or those who are unwilling to be fertilized, they may worry about the impacts of disease activity, medication history, and surgery on their fertility. Therefore, for CD women aged over 30 years and refusing fertilization, health education should be carried out to alleviate their anxiety about disease heritability and adverse pregnancy outcomes.

During the development of CD, pro-inflammatory factors are released into the blood, then penetrate the bowel wall, and trigger peritoneal inflammation that may further involve the ovaries. This pathology, though having not led to reproductive organic damage, may still decline fertility. Winger has found that the use of TNF-alpha inhibitors and intravenous immunoglobulin (IVIG) significantly improves in vitro fertilization (IVF) outcome in young infertile women withTh1/Th2 cytokine elevation [22]. In a study including 35 CD women, the AMH level is inversely associated with the score of Crohn’s Disease Activity Index (CDAI) [23]. In the present study, we found a consistent result that disease activity (OR, 4.314; 95%CI[1.561-12.910], *P* = 0.002) is an independent risk factor of decreased ovarian reserve. It has also proven that the risk of adverse pregnancy is higher in the active phase than in the remitting phase [20, 24]. Therefore, accelerating the disease into the remitting phase can reduce the damage to ovarian reserve and increase the odds of successful fertilization.

Some drugs have been suspected to have negative effects on ovarian reserve. In the present study, we reviewed the history of medication in CD women. Multivariate analysis discovered use of thalidomide (OR,12.628; 95%CI[4.351-42.380]; *P* < 0.001) as an independent risk factor for decreased ovarian reserve, which is consistent with our previous finding that thalidomide exerts negative effects on ovarian reserve [5]. Thalidomide, as a palliative and antiemetic, has been marketized to treat during-pregnancy vomiting, but later withdrawn for its teratogenic effect [25]. In 1960, its immunomodulatory effect was exploited to deal with Behcet’s disease and erythema nodosum [26]. Currently, this drug is selected for refractory CD that fails first-line and second-line treatments [27, 28].

We speculate that thalidomide can repress TGF to inhibit follicular growth and decrease ovarian reserve. TGF-α acts to promote the proliferation and differentiation of germ cells. In vitro studies have exhibited that TGF-α is expressed in ovarian follicles and theca-interstitial cells and regulates follicular growth, degeneration and atresia [29]. In the primordial phase, TGF-α is highly expressed in the oocytes, and as the primordial follicles develop, its expression decreases, indicating that it mainly modulates the development of primordial follicles and oocytes [30].

Thalidomide can suppress the expression of TNF-α, IL-6, IL-8, as well as TGF-α and TGF-β [31]. TGF downregulation further inhibits follicular growth, thus decreasing ovarian reserve. Therefore, CD women, if willing to be fertilized or pregnant, should avoid use of thalidomide. If unavoidable, their ovarian reserve should be closely monitored during medication.

We next assessed the effect of CD duration, extent, behavior and surgery on ovarian reserve. The results showed that they decreased ovarian reserve, but this decrease was not statistically significant. Previous studies have presented that surgery may decline the fertility of patients with inflammatory bowel disease, which can be explained by the fact that pelvic surgery, especially ileum pouch-anal anastomosis (IPPA), may injure reproductive organs, mainly the ovaries and oviducts [32]. But we did not find out the association between surgery and decreased ovarian reserve. We suppose that CD-related abdominal surgery brings less injury to pelvic organs in CD patients. In addition, the low fertility in CD women is not just associated with decreased ovarian reserve, but also post-surgical adhesions [33]. In the present study, we just evaluated the effect of ovarian reserve on fertility.

This study focused on the ovarian reserve function of patients with CD during their reproductive years, to explore the difference in AMH level between them and healthy women at matched age, and to analyze possible risk factors. Despite above-mentioned inspiring results, the present study, as a retrospective study, had certain limitations. First of all, although we recorded the patient’s fertility status, we lacked the data of adverse pregnancy outcome, and did not analyze the fertility status base on disease and drugs. Secondly, the methodology of serum AMH assays is still evolving and not globally standardized. The blood samples from both groups were not frozen during the study period and analyzed together at the end of recruiting for standardized AMH assay and methodology, which may cause some data bias. Finally, we did not compare AMH levels before and after medication. Long-term prospective studies may provide data on CD effect on ovarian reserve function in future. But we believe that our findings in the present study can be used to improve the management of CD women.

## Conclusion

The ovarian reserve in CD women of child-bearing age is lower than that in healthy controls. Age, disease activity and thalidomide use are risk factors of decreased ovarian reserve. Thalidomide should be avoided in the treatment of CD women desiring fertilization. To prevent age-related infertility, an active therapy should be administered to induce CD remission and reproductive health education is needed.

## Data Availability

The datasets used or analysed during the current study are available from the corresponding author on reasonable request.
